# Radiotherapy for Liver-Confined Hepatocellular Carcinoma in Elderly Patients with Comorbidity

**DOI:** 10.3390/cancers18010091

**Published:** 2025-12-27

**Authors:** Sun Hyun Bae, Young Seok Kim, Sang Gyune Kim, Jeong-Ju Yoo, Jae Myeong Lee, Sanghyeok Lim, Jae Hong Jung, Chan Kyu Kim

**Affiliations:** 1 Department of Radiation Oncology, Soonchunhyang University College of Medicine, Bucheon 14584, Republic of Korea; 2 Division of Gastroenterology and Hepatology, Department of Internal Medicine, Soonchunhyang University College of Medicine, Bucheon 14584, Republic of Korea; liverkys@schmc.ac.kr (Y.S.K.);; 3 Department of Radiology, Soonchunhyang University College of Medicine, Bucheon 14584, Republic of Korea; 4 Department of General Surgery, Soonchunhyang University College of Medicine, Bucheon 14584, Republic of Korea; 5 Division of Hematology-Oncology, Soonchunhyang University College of Medicine, Bucheon 14584, Republic of Korea

**Keywords:** Charlson Comorbidity Index, comorbidity, elderly, hepatocellular carcinoma, radiotherapy

## Abstract

**Background**: Globally, the incidence rate of hepatocellular carcinoma (HCC) has increased among elderly patients. Elderly patients often present with multiple comorbidities that affect treatment tolerance and outcomes, and the optimal management strategy for this population has not yet been established. Therefore, we assessed comorbidities in elderly patients and investigated the treatment outcomes of radiotherapy (RT) to liver-confined HCC. **Methods**: We retrospectively reviewed 40 elderly patients aged ≥70 years with liver-confined HCC, who were treated with RT between 2015 and 2023. Comorbidity was assessed by using the Charlson Comorbidity Index (CCI). Survival outcomes were analyzed using the Kaplan–Meier method. **Results**: The median age was 75 years (range, 70–87 years). The Barcelona Clinic Liver Cancer stage was 0 in 7 patients, A in 10 patients, B in 9 patients, and C in 14 patients. Most patients (85%) had Child–Pugh class A hepatic function before RT. The CCI scores ranged from 2 to 9 (median, 5). Various RT techniques were applied according to patients’ condition, tumor burden, and treatment aim: three-dimensional conformal radiotherapy in four patients, intensity-modulated radiotherapy in 20 patients; and stereotactic body radiotherapy in 16 patients. RT was delivered with radical intent in 30 patients and with palliative intent in 10 patients. The median biological effective dose calculated with an α/β ratio of 10 was 53.7 Gy_10_ (range, 39–134.4 Gy_10_). The median follow-up period after RT was 18 months. The 1-year local progression-free survival and overall survival (OS) rates were 74% and 81%, respectively, and the 3-year rates were 44% and 52%, respectively. Patients with CCI < 5 had more favorable OS than those with CCI ≥ 5, but the difference was not statistically significant. **Conclusions**: RT for liver-confined HCC appears to be a feasible treatment option for elderly patients with multiple comorbidities.

## Simple Summary

The incidence of hepatocellular carcinoma (HCC) is increasing among elderly populations, and its management in these patients has become a globally emerging issue. Elderly patients often present with multiple comorbidities that affect treatment tolerance and outcomes, and the optimal management strategy for elderly patients with HCC has not yet been established. This study is the first to assess comorbidities in elderly patients using the Charlson Comorbidity Index (CCI) and to investigate the treatment outcomes of radiotherapy (RT) for liver-confined HCC. RT appears to provide acceptable efficacy and low toxicity in elderly patients aged ≥70 years with comorbidities. CCI scores may be associated with survival and toxicity outcomes; however, further clinical studies are needed to confirm their clinical significance.

## 1. Introduction

Hepatocellular carcinoma (HCC) is the most common primary liver cancer and a major cause of cancer-related deaths worldwide [1]. The age-dependent nature of HCC risk is well known, with the median age at HCC diagnosis being 60 years [2]. As the global age structure continues to shift due to decreasing fertility rates and increasing life expectancy, the number of elderly patients diagnosed with HCC is expected to rise [3,4]. Defining “elderly” in HCC patients has become increasingly complex. The definition varies because life expectancy differs across geographic regions [5]. The World Health Organization typically defines old age as ≥65 years [6]. Some studies use thresholds of 75 or 80 years [7,8,9]. Most clinical studies on HCC, however, define elderly patients as those aged ≥70 years [10]. The International Society of Geriatric Oncology (SIOG) has reported that a cutoff point of 70 years is most commonly used for implementing geriatric assessments [11].

Effective management of elderly patients with HCC faces substantial challenges, primarily due to their higher susceptibility to concurrent comorbid conditions, which can hinder access to standard treatments [12]. Assessing comorbidities appears necessary for the appropriate management of these patients; however, there is ongoing debate regarding how to define and quantify the number and severity of associated comorbidities [13]. The Charlson Comorbidity Index (CCI) is one of the most widely used model for classifying comorbidities that may influence mortality risk [14]. It consists of 19 items, with higher scores indicating more severe comorbid conditions and, consequently, a greater risk of mortality. Several modified versions of the CCI have also been developed and applied. Although the CCI is extensively used in various cancers and among elderly patients, few clinical studies have investigated its impact on the clinical outcomes of HCC patients [15,16,17].

Radiotherapy (RT) is considered an attractive treatment modality for elderly patients with comorbidities, as it provides a noninvasive local therapy with limited systemic side effects [18]. Historically, the role of RT in HCC was restricted to palliative treatment. However, advances in RT techniques—such as three-dimensional conformal radiotherapy (3DCRT), intensity-modulated radiotherapy (IMRT), stereotactic body radiotherapy (SBRT), and particle therapy—along with an improved understanding of tumor biology and normal organ tolerance doses, have enabled RT to be applied for curative purposes [19]. Many clinical studies on the use of RT in the management of HCC have been reported [20,21,22]. The Barcelona Clinic Liver Cancer (BCLC) staging system, the most widely used algorithm for HCC management, released an updated version in 2025 that integrates RT into its treatment algorithm as an effective local modality [23]. However, data on the use of RT for HCC in elderly patients with comorbidities remain limited [24].

Therefore, we assessed comorbidities in elderly patients and investigated the treatment outcomes of RT for liver-confined HCC.

## 2. Materials and Methods

For this study, elderly patients were defined as those aged ≥70 years, considering that most previous studies have applied a cutoff ≥ 70 years for defining elderly patients and conducting geriatric assessment. Between April 2015 and December 2023, 53 elderly patients with HCC underwent RT at Soonchunhyang University College of Medicine, Bucheon. We retrospectively reviewed the patients’ medical records, and excluded 13 patients for the following reasons: (1) re-irradiation in the same patient to avoid duplication of data (n = 5); (2) transfer to another hospital resulting in loss of follow-up (n = 4); (3) foreign nationality with loss of follow-up (n = 2); and (4) presence of distant metastases (n = 2). The remaining 40 elderly patients aged ≥70 years who received RT for liver-confined HCC between April 2015 and May 2023 were included in this study. This study was approved by the Institutional Review Board (IRB) of Soonchunhyang University Bucheon Hospital (2025-05-003). A waiver of written informed consent was granted due to the retrospective nature of the study.

Comorbidity before RT was assessed using the CCI. Several modified versions have continued to be developed and published because the original CCI version may not be optimal for certain disease groups [25]. Therefore, the present study compared three versions of the CCI in elderly patients with HCC. The specific conditions included in the original CCI are summarized in Table 1. The age-adjusted CCI (ACCI) was calculated by adding age-related scores to the CCI (one score for each decade over 50 years) [15]. Lastly, the CCI excluding the liver disease condition (CCI-P) was also calculated [26].

All patients received RT using tomotherapy. Until February 2020, 17 patients underwent RT with the Hi-ART system. After the installation of Radixact in March 2020, 23 patients were treated with the Radixact. Details of the RT procedure have been described in our previous studies [27,28]. The treatment aim was categorized as either radical intent, in which all viable tumors were treated with RT, or palliative intent, in which only part of the tumors was treated with RT. Various RT techniques were selected according to patients’ condition, tumor burden, and treatment aim. Four patients received 3DCRT: portal vein tumor thrombosis (PVTT) only with 30 Gy in 10 fractions for palliative intent in two patients; PVTT and inferior vena cava tumor thrombosis (IVCTT) with 45 Gy in 25 fractions for palliative intent in one patient; and all viable tumors including HCC, PVTT, and IVCTT with 45 Gy in 25 fractions for radical intent in one patient. Twenty patients received IMRT: main mass ± PVTT with 33 Gy in 10 fractions for palliative intent in two patients; PVTT ± IVCTT with 30–44 Gy in 10–22 fractions for palliative intent in four patients; and main mass ± PVTT ± IVCTT with 40–60 Gy in 10–25 fractions for radical intent in 14 patients. Among the 16 patients treated with SBRT, only one patient received RT to an 8.8 cm HCC lesion among two lesions with 40 Gy in 4 fractions for palliative intent, while the remaining 15 patients received SBRT with 32–56 Gy in 4 fractions for radical intent. The median fraction size was 3.2 Gy (range, 1.8–4.0 Gy), and the median total dose was 44 Gy (range, 30–60 Gy). Because various fractionation schemes were used, the total doses were converted to the biologically equivalent dose (BED, Gy_10_) using the linear–quadratic model with an α/β ratio of 10.

Regular follow-up was performed 1–2 months after completion of RT and subsequently at 3-month intervals using computed tomography or magnetic resonance imaging. However, few patients underwent imaging studies at intervals of 4–6 months, either due to patient preference for longer intervals or because cation was required owing to decreased renal function. Local progression (LP) was defined as either progressive disease according to the modified Response Evaluation Criteria in Solid Tumors or as regrowth in any direction beyond that observed in pre-RT images of the treated lesions. Local-progression-free survival (LPFS) was calculated from the initiation date of RT to the date of LP or last follow-up. Intrahepatic progression-free survival (IHPFS) and overall survival (OS) were calculated from the initiation date of RT to the date of any intrahepatic tumor progression in the liver, and the date of death from any cause or last follow-up, respectively. In addition, HCC specific survival and non-HCC specific survival were calculated according to causes of death. Hepatic toxicity was defined as either classic radiation-induced liver disease (RILD)—characterized by anicteric hepatomegaly, ascites, or an alkaline phosphatase level exceeding twice the upper limit of the normal—or non-classic RILD, characterized by liver transaminases elevation exceeding five times the upper limit of the normal, or an increase in the Child–Pugh (CP) score by ≥2 points within 4 months after RT.

Among the three comorbidity indices, the optimal model was identified by calculating the area under the curve (AUC) of the receiver operating characteristics (ROC) curve. Survival outcomes were analyzed using the Kaplan–Meier method, and the log-rank test was used to compare prognostic factors with survival. All statistical analyses were conducted using the SPSS software version 26.0 (SPSS Inc., Chicago, IL, USA). Two-sided tests were used, and statistical significance was set at *p* < 0.05.

## 3. Results

The characteristics of the 40 elderly patients with liver-confined HCC are shown in Table 2. The median age of the patients was 75 years (range, 70–87 years). Most patients (70%) were male. Twenty-two patients (55%) had viral hepatitis. Liver cirrhosis was presented in 77%. Fourteen patients (35%) received RT as their initial treatment, while 26 patients (65%) had various courses of previous treatments. According to the BCLC staging system, 7 patients were assigned to the very early stage (0), 10 patients to the early stage (A), 9 patients to the intermediate stage (B), and 14 patients to the advanced stage (C). Thirteen patients (32%) received combined treatments during RT: concurrent hepatic arterial infusion chemotherapy in six patients, transarterial chemoembolization in three patients, atezolizumab and bevacizumab in two patients, sorafenib in one patient, and radiofrequency ablation in one patient. Thirty patients (75%) received RT with radical intent, while 10 patients (25%) received RT with palliative intent. The median BED was 55.6 Gy_10_ (range, 39.0–134.4 Gy_10_). All patients completed the planned RT schedule.

**Figure 1:**
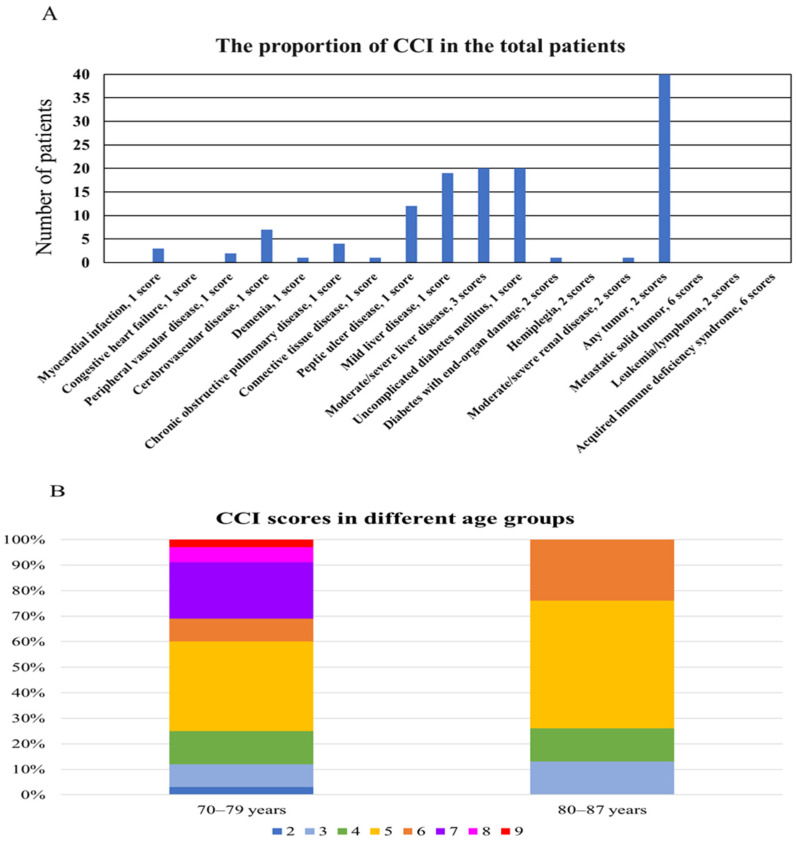
(**A**) Distribution of CCI conditions and scores in the total patients; (**B**) proportion of the Charlson comorbidity index (CCI) scores in different age groups.

Before RT, the median scores for the CCI, ACCI, and CCI-P were 5 (range, 2–9), 8 (range, 5–11), and 2 (range, 0–6), respectively. From ROC curve analysis, the AUC values for CCI, ACCI, and CCI-P were 0.620, 0.601, and 0.609, respectively. Therefore, CCI was selected as the model for assessing comorbidity. All patients had a score of 2 due to localized HCC, and only one patient did not have any other comorbidities (CCI score of 2). The common comorbidities, excluding the liver disease condition, were diabetes mellitus, peptic ulcer disease, and cerebrovascular disease (Figure 1A). CCI scores differed by age groups, as shown in Figure 1B. Patients in their seventies tended to have more comorbidities than patients in their eighties.

**Figure 2:**
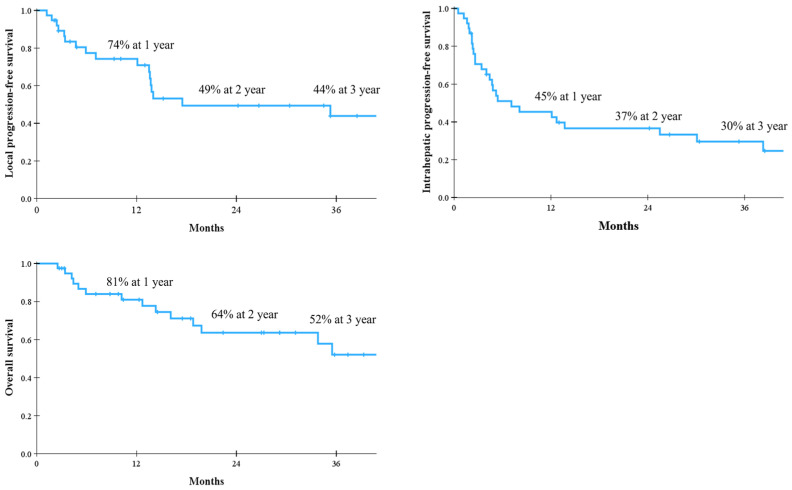
Survival after radiotherapy for hepatocellular carcinoma.

**Figure 3:**
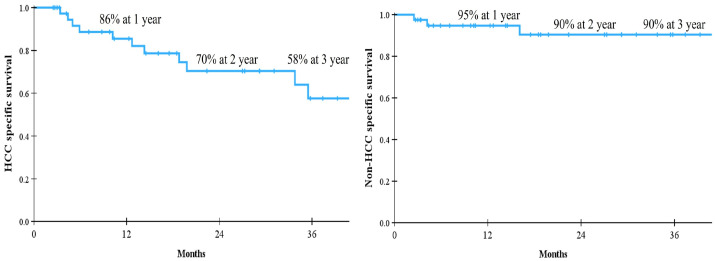
Survival according to causes of death in patients with hepatocellular carcinoma (HCC).

**Figure 4:**
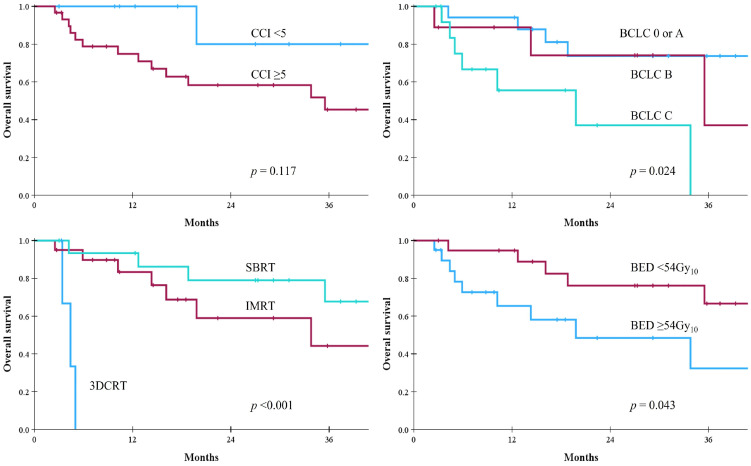
Univariate analysis on parameters affecting overall survival.

The median follow-up period after RT was 18 months (range, 3–85 months). The LPFS and IHPFS rates were 74% and 45% at 1 year, 49% and 37% at 2 years, and 44% and 30% at 3 years, respectively. The median OS was not reached and the 1-, 2-, and 3-year OS rates were 81%, 64%, and 52%, respectively. The corresponding survival curves are presented in Figure 2. When causes of death were analyzed, major cause of death was HCC-related in Figure 3. In the univariate analysis, baseline CP class, BCLC stage, PVTT, RT aim, RT technique, and BED were statistically significant parameters influencing both LPFS and IHPFS (Table 3). Among these, early stage, the absence of PVTT, radical treatment intent, the use of IMRT or SBRT, and a BED ≥ 54 Gy_10_ were significantly associated with improved OS. Patients with CCI scores < 5 had more favorable OS than those with CCI ≥ 5, but the difference was not statistically significant (Figure 4).

One patient (2%) experienced classic RILD but immediately recovered. She was followed for 51 months after SBRT without disease recurrence. Non-classic RILD occurred in eight patients (20%); among these, intrahepatic tumor progression was accompanied in six patients. The remaining two patients developed pneumonia that was unrelated to RT. One patient, a 76-year-old man with underlying chronic obstructive pulmonary disease and a CCI score of 10, received SBRT with 48 Gy in 4 fractions for a 2.8 cm HCC at segment 3/4. He experienced recurrent pneumonia involving both lungs, accompanied by deterioration of liver function, and died 4 months after RT. The other patient, an 83-year-old woman with dementia (CCI score of 10), received IMRT with 33 Gy in 10 fractions to 12 cm HCC at segment 5/6 for palliative intent. She developed pneumonia with worsening liver function and died 3 months after RT.

## 4. Discussion

To the best of our knowledge, this is the first study to evaluate comorbidities in elderly patients using three versions of the CCI and to investigate treatment outcomes of various RT techniques for liver-confined HCC. Among the CCI, ACCI, and CCI-P, the CCI showed the highest AUC value, with scores ranging from 2 to 9. This indicates that elderly patients generally have multiple comorbidities, yet all patients successfully completed the planned RT schedule. The 3-year LPFS, IHPFS, and OS rates for all patients were 44%, 30%, and 52%, respectively. Univariate analysis demonstrated that the use of SBRT was the most significant parameter (*p* < 0.001); the 3-year LPFS, IHPFS, and OS rates for elderly patients treated with SBRT were 73%, 53%, and 68%, respectively. The incidence of RILD after RT was low. Therefore, RT for liver-confined HCC appears to be a feasible treatment option for elderly patients aged ≥70 years with comorbidities.

There are distinct differences in the clinical characteristics of elderly patients compared with younger patients. First, elderly patients with HCC are more likely to have hepatitis C virus (HCV) infection, whereas younger patients more commonly have hepatitis B virus (HBV) infection [29]. In addition, elderly patients have a higher proportion of nonviral etiologies, including nonalcoholic fatty liver disease (NAFLD). HCV- and NAFLD-related HCC typically develop over a longer time than HBV-related HCC, leading to differences in disease etiology. Second, the proportion of females is higher among elderly patients, which may be attributed to women’s longer life expectancy [30]. Lastly, elderly patients tend to present with fewer HCC nodules than younger patients, with tumors in the elderly more often being well-differentiated, encapsulated, and associated with less vascular invasion [31]. However, HCC nodules in elderly patients are generally larger than those diagnosed in younger patients [32]. This may be related to the lack of regular HCC surveillance in patients without risk factors such as HBV or HCV infection, as well as to limited evidence supporting active HCC surveillance in elderly populations. In South Korea, HCC is the second leading cause of cancer-related deaths, and HBV infection remains the most common etiology (60% in 2022) [33]. Previous studies from our institution reflected this trend, showing that HBV-related hepatitis was the most frequent cause of underlying liver disease [27,28]. However, the current study demonstrated that 43% of elderly patients had HBV infection, whereas 45% had nonviral etiologies. The proportion of females was 30%, which was higher than that reported in other published studies. On the other hand, only 35% of patients received RT as their initial treatment, while the remaining patients underwent various prior treatments (1–16 sessions) before RT. Consequently, the BCLC stage distribution was variable.

Although the fit elderly patients who receive cancer treatment appear to derive benefits comparable to those of younger patients, age-related disparities in cancer care may arise from the difficulty of extrapolation of clinical trial results to real-world practice [34]. In this context, it remains important to verify whether RT in elderly patients is both effective and safe, even though RT for HCC is well established as a noninvasive local treatment modality. Only a few retrospective studies have focused specifically on elderly patients treated with RT for HCC, as summarized in Table 4 [35,36,37,38,39,40,41]. Earlier studies employed particle beam therapy, such as proton beam therapy (PBT) and carbon ion radiotherapy (CIRT). Theoretically, PBT and CIRT possess unique physical properties, characterized by the so-called Bragg peak—a finite range of tissue penetration followed by a sharp dose fall-off (a zero dose) beyond the beam path in tissues. This allows ablative doses to be delivered to tumors while minimizing radiation exposure to the normal liver, thereby reducing hepatic toxicity and improving therapeutic efficacy [22,42]. Although the “oldest-old” patients aged ≥80 years across with various disease stage were included, both PBT and CIRT showed superior survival outcomes compared with SBRT, as shown in Table 4.

LPFS and OS rates after SBRT in elderly patients were comparable with those reported in previously published SBRT studies [21]. But progression-free survival (PFS) rates after SBRT were approximately 30% at 3 years and appeared lower than historical data. Teraoka et al. [37] compared treatment outcomes of SBRT for ≤3 HCCs measuring ≤3 cm without macrovascular invasion between elderly patients (≥75 years) and younger patients (<75 years). Despite favorable LPFS and OS results, PFS in the elderly group was significantly inferior to that in the younger group (27% vs. 45% at 3 years; *p* = 0.03). The authors reported that elderly patients had a higher prevalence of HCV infection and suggested that aging might contribute to HCV-related hepatocarcinogenesis. Furthermore, aging itself may be a risk factor for recurrence after locoregional therapy in HCC [43]. Conversely, a recent study from a Chinese multicenter database reported that elderly patients (≥65 years) had a significantly lower recurrence rates and higher cause-specific survival than younger patients (<65 years) after hepatectomy [44]. Further clinical studies are warranted to clarify whether elderly patients with HCC have a higher risk of recurrence.

This study also included elderly patients treated with 3DCRT and IMRT. Among those treated with 3DCRT, all patients had PVTT and/or IVCTT and exhibited poor OS of 3–5 months. Although survival outcomes with IMRT were lower than those with SBRT, this difference reflected variations in baseline characteristics. For IMRT, the BCLC stage was 0 or A in 5 patients (25%), B in 5 patients (25%), and C in 10 patients (50%). In contrast, for SBRT, 12 patients (75%) were stage 0 or A, and four patients (25%) were stage B. The BED also differed: 39–78 Gy_10_ (median, 53.4 Gy_10_) for IMRT versus 57.6–134.4 Gy_10_ (median, 105.6 Gy_10_) for SBRT. Although a dose–response relationship in HCC has not been firmly established, recent evidence highlights the clinical relevance of achieving a sufficient minimum dose in RT for HCC. The American Society for Radiation Oncology clinical practice guideline suggested that dose escalation to a minimum BED of 65–100 Gy_10_ may provide improved local control in HCC [19]. Furthermore, recent expert consensus recommends a minimum BED ≥ 80 Gy_10_ to achieve an ablative effect in HCC [45]. The higher survival observed with SBRT in this study may be attributed to its higher BED compared with 3DCRT or IMRT. Generally, IMRT is applied in advanced HCC with larger tumor size and shows similar survival outcomes with 3DCRT, while the incidence of RILD remains relatively low [20]. In addition, respiratory control during SBRT is often challenging in elderly patients [40]. Potential dosimetric errors induced by respiratory motion between the planned RT dose and delivered RT doses tend to average out through fractionation, supporting the safe use of IMRT when irregular respiration in elderly patients cannot be adequately corrected [46].

Two studies have reported comorbidity assessments in elderly patients treated with RT for HCC [39,41]. Loi et al. [39] analyzed the ACCI and Geriatric 8 (G8) scores in 42 elderly patients aged ≥80 years who received SBRT for HCC. The G8 tool was originally developed to identify elderly patients (>70 years) with various cancers who would benefit from a comprehensive geriatric assessment prior to first-line chemotherapy [47]. It consists of eight components: age category (>85, 80–85, or <80 years), and seven items from the Mini Nutritional Assessment (MNA) questionnaire, including appetite changes, weight loss, mobility, neuropsychological problems, body mass index, medication, and self-rated health status. The median ACCI and (G8) scores were 10 (range, 7–16) and 11 (range, 8–14), respectively. The authors found that a G8 score > 10 significantly affected improved survival (*p* = 0.045), whereas an ACCI score ≥ 10 was correlated with increased acute toxicity (*p* = 0.021). Another study evaluated G8 scores in 24 elderly patients aged ≥70 years who received SBRT for HCC and reported that patients with G8 scores > 9 had significantly improved survivals (*p* < 0.001) [41]. In Sweden, a national registry study examined non-liver comorbidity (CCI-P) in 980 patients with HCC (mean age, 65 years) who received curative treatments [26]. The CCI-P scores were strongly associated with age and were highest among patients who underwent ablation and lowest among those who received liver transplantation (*p* < 0.001). Additionally, CCI-P scores were significantly associated with 5-year mortality. In the present study, three comorbidity indices—CCI, ACCI, and CCI-P—were used to assess comorbidities in elderly patients. Among these, CCI was the most predictive tool, although the AUC value was modest (0.620). Patients in their seventies exhibited more comorbidities than those aged ≥80 years (Figure 1B), possibly because RT was selectively considered for the oldest-older patients ≥ 80 years with good general condition. Although patients with CCI < 5 had more favorable OS, the difference was not statistically significant. Comorbidities in elderly patients may influence survival; however, discrepancies among studies should be clarified through further clinical research. Regarding toxicity, two patients (CCI scores of 10 and 10; ACCI scores of 11 and 12) developed pneumonia unrelated to RT and subsequently died. Considering the finding of this study and Loi et al. [39], underlying comorbidities in elderly patients may deteriorate unpredictably, and caution is warranted when applying RT to elderly patients with high CCI scores.

Our study has several limitations. First, this study was a retrospective study; therefore, selection bias may have occurred. Our institution is a tertiary medical center where patients primarily visit for cancer care. In Korea, the management of comorbidities is mainly provided in primary and secondary healthcare settings, and some individual diagnoses of CCI conditions may have been missed. However, since patients admitted for initial HCC evaluation routinely undergo assessment of underlying comorbidities, the likelihood of missing data may have been minimized. Second, the sample size was small, and the follow-up period was not sufficiently long. Although several clinical parameters were significantly associated with survival in the univariate analysis, subgroup analyses could not be performed because of the limited sample size. We identified several noteworthy findings related to the CCI, but additional statistical analyses could not be conducted. Verification of these results is required in future clinical studies with larger patient cohorts with long-term follow-up. Third, we used a single tool—the CCI—to assess comorbidities. Although the CCI is simple to complete, is highly suitable for cohort studies, and can be derived from hospital records, the most appropriate index for comorbidity assessment among several available tools has not yet been determined [48]. Further research is warranted to compare diverse indices and identify the most effective tool for evaluating comorbidity in patients with HCC. Lastly, our study included various RT techniques, including 3DCRT, IMRT, and SBRT. Because 3DCRT or IMRT is typically used in elderly patients who are not suitable candidates for SBRT, treatment outcomes in this study appear lower than those reported in other studies summarized in Table 4. However, in subgroup analysis, survival outcomes following SBRT were comparable to those reported in previous research. For cases involving large HCCs, normal liver volume < 1000 mL, or poor patient compliance during RT, 3DCRT or IMRT may be more appropriate than SBRT. The optimal RT technique will be carefully selected based on patients’ overall condition and tumor burden.

## 5. Conclusions

RT for liver-confined HCC appears to provide acceptable efficacy and low toxicity in elderly patients aged ≥70 years with comorbidities. CCI scores may be associated with both survival and treatment-related toxicities; however, further clinical studies would be needed to confirm their clinical significance.

## Figures and Tables

**Table 1:** Charlson Comorbidity Index.

Score	Condition
1	Myocardial infarction, congestive heart failure, peripheral vascular disease, cerebrovascular disease, dementia, chronic obstructive pulmonary disease, connective tissue disease, peptic ulcer disease, mild liver disease ^a^, uncomplicated diabetes mellitus
2	Hemiplegia, moderate to severe renal disease, diabetes with end-organ damage, any tumor, leukemia, lymphoma
3	Moderate/severe liver disease ^b^
6	Metastatic solid tumor, acquired immune deficiency syndrome

^a^ mild = chronic hepatitis or cirrhosis without portal hypertension. ^b^ moderate = cirrhosis and portal hypertension but no variceal bleeding history; severe = cirrhosis and portal hypertension with variceal bleeding history.

**Table 2:** Patients’ characteristics.

Parameters		No. of Patients	Parameters		No. of Patients
Age	Median 75 years (70–87 years)		Baseline CP class	A	34 (85%)
Sex	Male	28 (70%)		B	6 (15%)
	Female	12 (30%)	BCLC stage	0	7 (17%)
CCI	Median 5 (2–9)			A	10 (25%)
ACCI	Median 8 (5–11)			B	9 (23%)
CCI-P	Median 2 (0–6)			C	14 (35%)
Viral hepatitis	No	18 (45%)	PVTT	No	27 (68%)
	HBV	17 (43%)		Yes	13 (32%)
	HCV	4 (10%)	Combined treatment	No	27 (68%)
	HBV/HCV	1 (2%)		Yes	13 (32%)
Liver cirrhosis	No	9 (23%)	RT aim	Radical intent	30 (75%)
	Yes	31 (77%)		Palliative intent	10 (25%)
Previous treatment	No	14 (35%)	RT technique	3DCRT	4 (10%)
	Yes	26 (65%)		IMRT	20 (50%)
Type of previous treatment	Surgery	11 (28%) (1–2 cycles)		SBRT	16 (40%)
	RFA	3 (8%) (1–2 cycles)	GTV	Median 32.7 cc (2.3–1241.8 cc)	
	TACE	23 (57%) (1–16 cycles)	PTV	Median 143.8 cc (17.8–1818.0 cc)	
	RT	1 (2%)	Fraction size	Median 3.2 Gy (1.8–14.0 Gy)	
	Sorafenib	1 (2%)	Total dose	Median 44 Gy (30–60 Gy)	
	Ate/beva	1 (2%)	BED	Median 55.6 Gy_10_ (39.0–134.4 Gy_10_)	

Abbreviations: ACCI—age-adjusted Charlson comorbidity index; CCI-P—CCI excluding liver disease and hepatocellular carcinoma; HBV—hepatitis B virus; HCV—hepatitis C virus; RFA—radiofrequency ablation; TACE—transarterial chemoembolization; Ate/beva—combined treatment with atezolizumab and bevacizumab; CP—Child–Pugh; BCLC—Barcelona Clinic Liver Cancer; PVTT—portal vein tumor thrombosis; RT—radiotherapy; 3DCRT—3-dimensional conformal radiotherapy; IMRT—intensity-modulated radiotherapy; SBRT—stereotactic body radiotherapy; GTV—gross tumor volume; PTV—planning target volume; BED—biologically effective dose when α/β ratio was assumed to be 10 Gy.

**Table 3:** Univariate analysis for parameters affecting local progression-free survival (LPFS), intrahepatic progression-free survival (IHPFS), and overall survival (OS).

Parameters		1Y LPFS	3Y LPFS	*p*-Value	1Y IHPFS	3Y IHPFS	*p*-Value	1Y OS	3Y OS	*p*-Value
Age	<80 years	72%	41%	0.509	41%	26%	0.284	79%	54%	0.907
	≥80 years	83%	63%		67%	50%		88%	37%	
Sex	Male	70%	38%	0.140	37%	24%	0.126	76%	64%	0.704
	Female	82%	58%		64%	41%		92%	43%	
CCI	<5	89%	59%	0.513	40%	40%	0.905	100%	80%	0.117
	≥5	69%	39%		47%	25%		75%	45%	
Baseline CP class	A	79%	49%	0.011	51%	33%	0.014	84%	56%	0.066
	B	44%	0%		17%	-		60%	-	
BCLC stage	O/A	94%	86%	<0.001	74%	58%	<0.001	94%	74%	0.024
	B	86%	21%		51%	25%		89%	37%	
	C	41%	0%		8%	0%		56%	0%	
PVTT	No	92%	63%	<0.001	63%	44%	<0.001	92%	67%	0.005
	Yes	35%	0%		8%	0%		51%	0%	
RT aim	Radical	89%	55%	<0.001	58%	38%	<0.001	93%	59%	0.001
	Palliative	26%	0%		0%	0%		26%	-	
RT technique	3DCRT	-	-	<0.001	0%	0%	<0.001	0%	0%	<0.001
	IMRT	66%	22%		39%	14%		83%	44%	
	SBRT	93%	73%		61%	53%		93%	68%	
BED	<54 Gy_10_	53%	13%	<0.001	28%	11%	0.002	65%	32%	0.043
	≥54 Gy_10_	94%	75%		62%	47%		95%	7%	

Abbreviations: CCI—Charlson comorbidity index; CP—Child–Pugh; BCLC—Barcelona Clinic Liver Cancer; PVTT—portal vein tumor thrombosis; RT—radiotherapy; 3DCRT—3-dimensional conformal radiotherapy; IMRT—intensity-modulated radiotherapy; SBRT—stereotactic body radiotherapy; BED—biologically effective dose when α/β ratio was assumed to be 10 Gy; Y—year.

**Table 4:** Clinical studies for elderly patients with hepatocellular carcinoma treated with radiotherapy (RT).

Authors (Year)	Age (Years) (Median)	No. of Pts	Indication	RT Technique	Median RT Dose (Gy) (Range)	No. of Fractions	LPFS	PFS	OS	RILD
Hata [35] (2007)	80–85 (81)	21	UICC T1 or T3	PBT	66 (60–70)	10–35	100% at 1Y	70% at 1Y	84% at 1Y	0%
							100% at 3Y	51% at 3Y	62% at 3Y	
Shiba [36] (2017)	80–95 (83)	31	BCLC A–C	CIRT	60 (52.8 or 60)	4 or 12	89% at 2Y	51% at 2Y	82% at 2Y	3%
Teraoka [37] (2018)	75–93 (79)	54	≤3 HCCs ≤ 3 cm, MVI (-)	SBRT	48 (40, 48, 60)	4 or 8	100% at 1Y	51% at 1Y	96% at 1Y	NR
							98% at 3Y	27% at 3Y	64% at 3Y	
Iwata [38] (2021)	80–96 (82)	71	BCLC 0–D	PBT	66 (66 or 72.6)	10 or 22	88% at 2Y	50% at 2Y	76% at 2Y	0%
Loi [39] (2021)	80–91 (85)	42	BCLC A or B	SBRT	54 (30–75)	3–10	93% at 1Y	47% at 1Y	72% at 1Y	0%
							93% at 2Y	31% at 2Y	43% at 2Y	
Jang [40] (2022)	75–90 (77)	83	≤2 HCCs ≤ 6 cm, MVI (-)	SBRT	45 (36–60)	3–4	98% at 3Y	30% at 3Y ^a^	57% at 3Y	NR
							90% at 5Y	10% at 5Y ^a^	41% at 5Y	
Sharma [41] (2025)	70–84 (75)	24	BCLC C	SBRT	35 (25–40)	5	90% at 1Y	42% at 1Y	58% at 1Y	0%
Current study	70–87 (75)	40	BCLC 0–C	Overall	44 (30–60)	4–25	74% at 1Y	45% at 3Y ^a^	81% at 1Y	2%
							44% at 3Y	30% at 3Y ^a^	52% at 3Y	
				3DCRT	37.5 (30 or 45)	10 or 25	-	0% at 3Y ^a^	0% at 3Y	
				IMRT	44 (30–60)	10–25	22% at 3Y	14% at 3Y ^a^	44% at 3Y	
				SBRT	48 (32–56)	4	73% at 3Y	53% at 3Y ^a^	68% at 3Y	

Abbreviations: UICC—International Union Against Cancer staging; T—tumor; BCLC—Barcelona Clinic Liver Cancer staging; MVI—macrovascular invasion; PBT—proton beam therapy; CIRT—carbon ion radiotherapy; SBRT—stereotactic body radiotherapy; 3DCRT—3-dimensional conformal radiotherapy; IMRT—intensity-modulated radiotherapy; LPFS—local progression-free survival; PFS—progression-free survival; OS—overall survival; RILD—radiation-induced liver disease; NR—not reported. ^a^ means intrahepatic progression-free survival.

## Data Availability

The datasets generated and/or analyzed in this study are available from the corresponding author due to ethical reasons.
